# AGE DIFFERENCES IN SUNK COST DECISIONS FOR ONESELF VERSUS A LOVED ONE

**DOI:** 10.1093/geroni/igad104.0096

**Published:** 2023-12-21

**Authors:** JoNell Strough, Wändi Bruine de Bruin, Andrew Parker

**Affiliations:** WVU, Morgantown, West Virginia, United States; University of Southern California, Los Angeles, California, United States; RAND Corporation; Pardee RAND Graduate School, Santa Monica, California, United States

## Abstract

Previous research has established that older adults are less biased by irretrievable prior investments or ‘sunk costs’ when making a decisions about continuing a failing project. Such decisions often have implications for other people, but most research to date has focused on individuals making decisions for themselves. Here, we investigated whether age differences in willingness to discontinue a failing project and start over depended upon the intended beneficiary (self or loved one) as well as the type of investment (money or time). Participants (N=1075, Mage=53.49 yrs; 56.2% Women) from the American Life Panel responded to an online survey and made hypothetical decisions about a failing project. At all ages, when the project was for oneself, individuals were less willing to discontinue it and start over when money (versus time) had been invested, whereas when the project was for a loved one, willingness to start over did not depend on whether money or time had been invested. The association between older age and greater willingness to discontinue a failing project and start over was stronger when projects were for oneself compared to a loved one. Implications of the findings for understanding aging and decision making in a social context, and strategies for reducing sunk-cost bias in people of all ages are discussed.

